# A Rare Encounter with an Expanding Pseudocyst of the Spleen

**DOI:** 10.1155/2017/9896856

**Published:** 2017-12-25

**Authors:** Ashish Lal Shrestha, Pradita Shrestha

**Affiliations:** Department of General Surgery, United Mission Hospital, Tansen, Palpa, Nepal

## Abstract

**Background:**

Splenic Pseudocyst (SP) is a diagnostic rarity, with cystic lesions of spleen themselves being uncommon. Establishing a preoperative diagnosis could help in specific management but this is rather challenging. Here we present a common presentation of an uncommon diagnosis.

**Case Presentation:**

A 47-year-old lady, previously well, presented to the outpatient clinic with intermittent left hypochondrial pain radiating towards left shoulder for 2 months not associated with fever, jaundice, or weight loss. Abdominal examination revealed nontender hepatosplenomegaly. The initial abdominal ultrasonogram (USG) was suggestive of a hydatid cyst, for which she received a course of antihelminthics. At follow-up, after finding no clinical improvement and radiological worsening, she underwent an exploratory laparotomy. A cyst replacing entire lower pole and a significant portion of splenic hilum was found. Total splenectomy was performed. The specimen was reported to be a SP.

**Conclusion:**

SP is a unique entity, usually misdiagnosed as a parasitic lesion and often treated with antihelminthic medicines. The natural course of disease, however, follows a subsequent failure of symptom resolution and radiological worsening that ultimately demands surgical attention. Based on size, location, and intraoperative findings, either total or partial splenectomy is required. The final histopathological report often presents a diagnostic surprise.

## 1. Introduction

Cysts in the spleen are uncommon, and amongst these one rare kind is SP [1, 2]. Previous blunt abdominal trauma is implicated in at least 75% of cases [1, 3].

We report an interesting case of a rare and expanding SP without history of previous abdominal trauma. The clinical presentations, investigative findings, and management are discussed with relevant literature review.

The rarity of this case lies in the fact that it is often misdiagnosed and wrongly treated and eventually requires surgical exploration [4]. Unless a high degree of clinical suspicion is maintained, it is likely to be missed and may result in complications that may be fatal at times.

The peroperative findings and final histopathological report usually take the surgeon by surprise.

## 2. Case Presentation

A 47-year-old lady without significant past medical or surgical history presented to the outpatient clinic with intermittent episodes of left hypochondrial pain radiating towards the left shoulder for 2 months. She did not have associated fever, jaundice, or altered bowel habits. Some loss of appetite was noted without loss of weight. She could not recall any abdominal trauma in the recent past. Physical examination was unremarkable except for a nontender hepatosplenomegaly.

Hematological and biochemical tests were normal. Abdominal radiographs were unremarkable. Abdominal USG revealed a complex cystic lesion in the spleen measuring 4 × 8 × 6 cm with poorly defined double wall and multiple internal septations suggesting a likely hydatidosis along with enlarged fatty liver.

She was given a course of antihelminthics, vaccinated against capsulated organisms predicting possibility of a subsequent splenectomy, and asked to return a month later.

**Figure 1:**
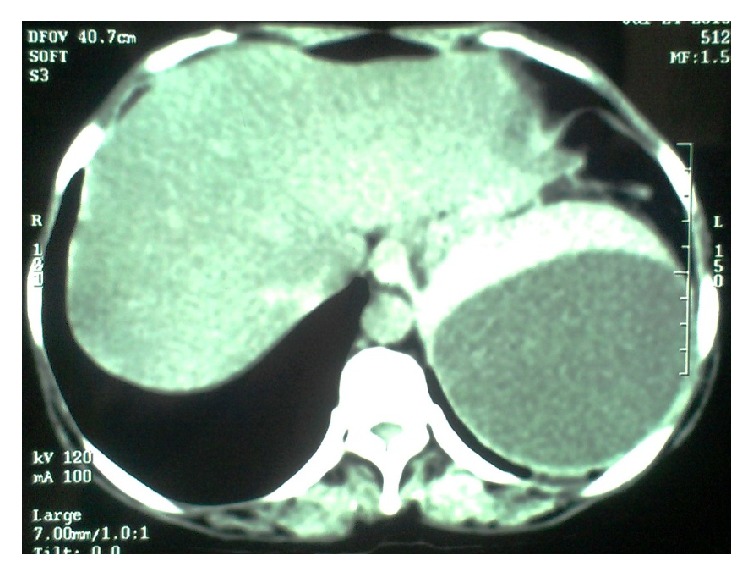
CECT scan of the abdomen showing a nonenhancing cystic lesion arising from lower pole and hilum of spleen measuring 10 × 9 × 9 cm with multiple internal septations and abutting the tail of pancreas.

At follow-up, her symptoms persisted and repeated abdominal USG showed expanding splenic cyst measuring 9 × 8 × 8 cm. CECT scan of the abdomen showed a nonenhancing cystic lesion arising from lower pole and hilum of spleen measuring 10 × 9 × 9 cm with multiple internal septations and abutting the tail of pancreas as shown in Figure 1.

A list of differentials was considered that included a splenic hydatid cyst, pancreatic tail pseudocyst, and a mesenteric cyst. In view of clinical and radiological worsening, she was taken for an elective exploration through a left subcostal incision.

**Figure 2:**
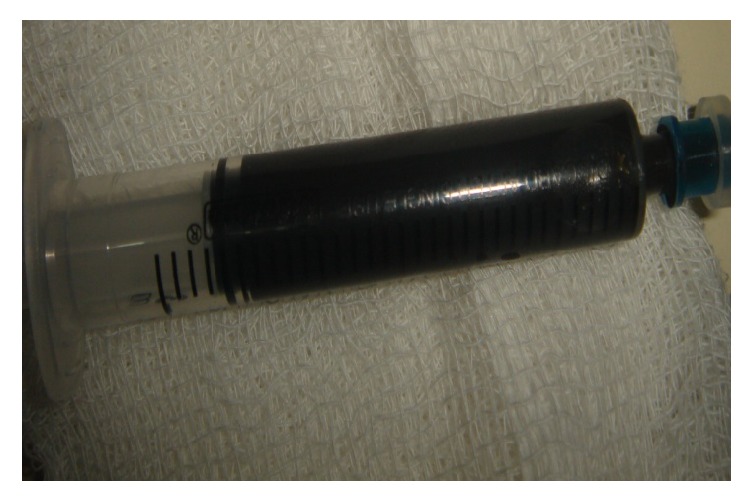
The brownish translucent fluid aspirated from the expanding splenic cyst, the routine bacterial culture of which was later reported to be sterile.

At laparotomy, a huge splenic cyst was found occupying entire lower pole and significant portion of hilum and measuring 9 × 9 × 8 cm. The aspiration of this revealed a brownish translucent fluid as shown in Figure 2, the routine bacterial culture of which was later reported to be sterile.

**Figure 3:**
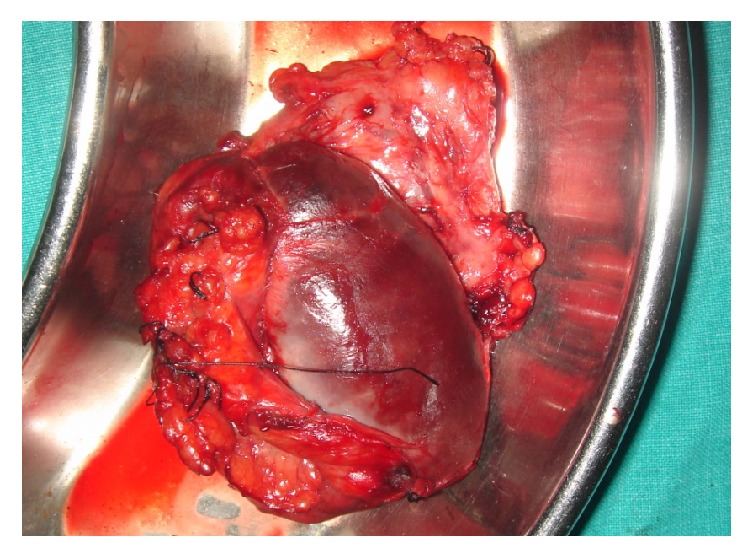
The intraoperative image of the splenic cyst causing pressure atrophy of the residual splenic parenchyma. During intraoperative manipulation, the cyst wall was inadvertently ruptured.

The cyst was causing pressure atrophy of the residual splenic parenchyma and also had multiple dense perisplenic and pericystic adhesions. Total splenectomy was done. During intraoperative manipulation, the cyst wall got inadvertently ruptured. The image of splenectomy specimen is shown in Figure 3.

She had an uneventful postoperative recovery and was discharged on the 7th postoperative day. At 2-week and one-year follow-up, she remained symptom-free.

Histopathologically, gross examination confirmed the operative findings and showed a unilocular already cut-open cyst measuring 9 × 5 cm with wall thickness measuring 2–5 mm.

**Figure 4:**
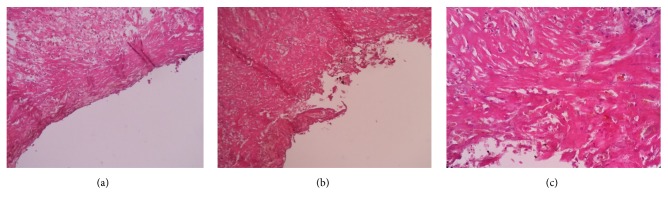
Microscopic appearance of the Splenic Pseudocyst (stained with Eosin/Hematoxylin stain) at (a) 10x magnification, (b) 25x magnification, (c) 40x magnification.

Microscopic section showed a cyst wall that was composed of hyalinized fibrous tissue without epithelial lining. Also noted were plenty of extravasated red blood cells and hemosiderin laden macrophages over the cyst wall. There was no evidence of cellular atypia. The microscopic image of the SP is shown in Figure 4.

The features confirmed a SP.

## 3. Discussion

Splenic cysts have been a matter of curiosity, with a reported incidence of only around 800 globally [1, 5–7]. Since their recognition in 1829 by Andral and first splenectomy in 1867 by Pean for this condition, there have been infrequent reports in the literature [8]. The earlier system classified these lesions into type 1 (true cysts with lining epithelium) and type 2 (false cysts without lining epithelium) [9–12]. A consecutive modification divided these into parasitic and nonparasitic varieties, further categorizing nonparasitic ones into primary (epithelial/true) and secondary (false/pseudo) types [13]. Parasitic ones follow a geographical distribution and account for more than 2/3rds of cases in the endemic areas [8]. Of these, the commonest etiology is* Echinococcus granulosus* [8]. This holds true even in the clinical context of Nepal [14]. More recently, a pathological classification was suggested dividing nonparasitic cysts into congenital, traumatic, neoplastic, and degenerative types [10, 11].

Of all the cysts, SPs constitute 70–80%, particularly affecting women, children, and young adults [1, 4, 8, 15]. The incidence can be expected to rise further with nonoperative management of blunt abdominal trauma becoming more popular.

In symptomatic 2/3rds, symptoms include left hypochondrial pain radiating to the left shoulder or chest [5, 8, 16]. Other symptoms include early satiety, vomiting, dysphagia, and infrequently ipsilateral atelectasis and lower lobe pneumonia depending upon the location and organ of compression [1, 17]. Symptoms also depend on the size of the SP which forms the basis for operative treatment and predicting complications. While SP larger than 5 cm usually dictates operative management, the risk of complications, like sudden increase in size due to intracystic bleed, secondary infections, and even fatality due to spontaneous intraperitoneal rupture, has been noted in larger SP [1, 5, 18]. SP may sometimes attain great dimensions with those exceeding 15 cm entitled giant pseudocysts [4, 11, 19, 20].

SP may also be incidental sonological finding or detected due to calcification on radiographs [17]. USG, CECT, MRI, and MRA can all help to delineate cystic nature of the lesion [4, 8]. However, the precise preoperative radiological diagnosis remains challenging, although it could be a great aid to efficient and specific management.

Most often, misdiagnosed as parasitic lesions, these cysts are often treated with antihelminthics only to find unsatisfactory response and radiological deterioration at follow-up. Our patient had a similar treatment course.

Etiologically, SP represents a resolved hematoma in the parenchymal or subcapsular location due to a preceeding blunt injury [1, 2, 4]. Suggested alternate etiologies include infections and degenerative diseases [4, 21].

In gross appearance, majority of these are unilocular and smooth walled while microscopic findings consist of fibrous wall tissue without an epithelial lining [4, 5, 7, 8, 19].

Traditional approach to SP larger than 5 cm has been total splenectomy. However, with growing knowledge about protective role of spleen as an organ of reticuloendothelial and hematopoietic importance, more specifically in terms of OPSI, the current approach has been of splenic conservation [4, 7, 22]. In this regard, partial splenectomy, cyst aspiration, deroofing, marsupialisation, decapsulation, and cystectomy have all been described by both open and laparoscopic routes [4, 20, 22, 23]. Laparoscopic unroofing and drainage have been found to have a recurrence rate of 20–40%, and hence to avoid this, marsupialisation or decapsulation has been the recommended technique [18, 24].

Certain characteristics of SP like hilar location, large size with near complete replacement of parenchyma, associated hypersplenism, and doubtful diagnosis are the few important situations where total splenectomy may not be avoidable [7, 22, 25].

In our patient, the likelihood of hydatid etiology was considered earlier in view of endemicity of infestation and hence was managed in similar lines. There was no way to prove or disprove this diagnosis, and the much talked about “Casoni's intradermal test” was also unavailable in a rural set-up like ours.

At follow-up, since no clinical improvement was found predicting a possibility of life threatening complication like rupture or hemorrhage in future, elective exploration was considered. This was supported with evidence of expanding cyst dimensions and hilar location that made the decision of total splenectomy rather simple. Following this, the patient had an uneventful recovery and remained symptom-free at 1-year follow-up.

## 4. Conclusions

In conclusion, SP is uncommon pathology that is capable of mimicking commoner conditions like hydatidosis. The clinical and radiological pictures may be frequently misleading with consequent mismanagement. A high degree of clinical suspicion is, therefore, as essential as the understanding of potential complications to avoid clinical mishaps.

Definitive diagnosis is possible only on histopathology and usually poses a diagnostic surprise. However, once treated adequately, SP has a good outcome. Hence, awareness of its clinical presentation and good pathological expertise are important adjuncts in the diagnosis.

## Abbreviations

SP: Splenic Pseudocyst
USG: Ultrasonogram
CECT: Contrast enhanced computed tomography
MRI: Magnetic resonance imaging
MRA: Magnetic resonance arteriogram
OPSI: Overwhelming postsplenectomy infections.
